# IOUC-3DSFCNN: Segmentation of Brain Tumors via IOU Constraint 3D Symmetric Full Convolution Network with Multimodal Auto-context

**DOI:** 10.1038/s41598-020-63242-x

**Published:** 2020-04-10

**Authors:** Jinping Liu, Hui Liu, Zhaohui Tang, Weihua Gui, Tianyu Ma, Subo Gong, Quanquan Gao, Yongfang Xie, Jean Paul Niyoyita

**Affiliations:** 1 Hunan Provincial Key Laboratory of Intelligent Computing and Language Information Processing, Hunan Normal University, Changsha, Hunan 410081 China; 2 School of Automation, Central South University, Changsha, Hunan 410083 China; 3 Department of Geriatrics, The Second Xiangya Hospital of Central South University, Changsha, 410011 China; 4 College of Science and Technology, University of Rwanda, Kigali, 3286 Rwanda

**Keywords:** Cancer imaging, Image processing

## Abstract

Accurate segmentation of brain tumors from magnetic resonance (MR) images play a pivot role in assisting diagnoses, treatments and postoperative evaluations. However, due to its structural complexities, e.g., fuzzy tumor boundaries with irregular shapes, accurate 3D brain tumor delineation is challenging. In this paper, an intersection over union (IOU) constraint 3D symmetric full convolutional neural network (IOUC-3DSFCNN) model fused with multimodal auto-context is proposed for the 3D brain tumor segmentation. IOUC-3DSFCNN incorporates 3D residual groups into the classic 3DU-Net to further deepen the network structure to obtain more abstract voxel features under a five-layer cohesion architecture to ensure the model stability. The IOU constraint is used to address the issue of extremely unbalanced tumor foreground and background regions in MR images. In addition, to obtain more comprehensive and stable 3D brain tumor profiles, the multimodal auto-context information is fused into the IOUC-3DSFCNN model to achieve end-to-end 3D brain tumor profiles. Extensive confirmatory and comparative experiments conducted on the benchmark BRATS 2017 dataset demonstrate that the proposed segmentation model is superior to classic 3DU-Net-relevant and other state-of-the-art segmentation models, which can achieve accurate 3D tumor profiles on multimodal MRI volumes even with blurred tumor boundaries and big noise.

## Introduction

As a common and high-risk disease, brain tumor is an abnormality in brain tissues, leading to severe damage to the nervous system. Among brain tumors, glioma accounts for almost 80% primary malignant tumors with the highest fatal rate due to its quick progression^1^. Despite considerable effort in glioma research, the survival rate of glioma patients is still low^2^. Given the high fatal nature and rapid growth of glioma, early diagnosis of glioma is crucial for better clinical treatment planning that can help to increase the survival rate and improve the life quality of patients.

Magnetic resonance imaging (MRI), as a powerful non-invasive analysis tool, has been applied to the imaging diagnosis of various systems throughout the body^1,3^. By qualitative or quantitative analysis of brain tumors in MR images, physicians can achieve key information, such as location, size, shape, irregularity, intra-tumoral structure of brain tumors^4^ to identify the growth state of brain tumors and evaluate the success of a chosen treatment strategy^2^. Due to the large volume of MR images, manual brain tumor segmentation are time-consuming and prone to human errors, limiting the precisely quantitative measurements and malignancy evaluation of brain tumors in the clinical practice^5^.

Therefore, designing an accurate, highly-efficient and reliable segmentation method for the automated brain tumor segmentation of MR images attracted world-wide attention^6,7^, leading to a wealth of techniques for the automated, semi-automated, or interactive segmentation of brain tumors. As summarized in Menze’s work^2^, these methods can be categorized into two families, the generative methods and discriminative methods.

Generative methods establish explicit parametric or non-parametric (probabilistic) models for different tissues by incorporating domain-specific prior information, e.g., the appearance and spatial distribution of different tissues, and tumors are generally delineated as outliers of normal tissues. Generative methods offer the advantage of easy incorporation of prior information, thus they exhibit good generalization to unseen images and achieved the state-of-the-art results on many brain tissue segmentation tasks in earlier years^8,9^. However, achieving explicit models of anatomies and appearances of different tissues is often problematic due to the unpredicted variations of shapes, sizes, locations of tumors in MR image intensities^2^, which decreases the reliabilities of generative methods.

Methods directly learn a mapping model between low level MR image intensity features and segmentation labels without exploiting domain knowledge. Typical discriminative methods usemachine learning models, such as support vector machines^10,11^, decision tree^12^ or random forest^13^, with elaborate dense, voxel-wise MR image features, such as statistics from local intensity differences or co-occurrence matrixes^10^, and intensity distribution characteristics^14,15^, and so on. However, designing an ideal handcraft feature descriptor for the effective characterization of highly variable tumors is labor intensive and experience dependent, limiting the practical applications of the discriminative methods.

## FCNN-based brain tumor segmentation

In recent years, deep learning models, with convolution neural networks (CNNs), have revolutionized computer vision through end-to-end learning, i.e., integrating automated features learning from large-scale raw image data with supervised classifier, attracting increasing interest in brain tumor segmentation, which can achieve the state-of-art segmentation result^6^. For instance, Pereira *et al*.^5^ proposed a deep CNN-based automatic glioma segmentation model with small convolutional kernels; Hu *et al*.^16^ combined multi-cascaded CNN with fully connected conditional random fields (CRFs) for the brain tumor segmentation.

Due to the commonly-existing limitations of deep CNN-based segmentation in sliding-windowed dense pixel label prediction, such as limited receptive filed, high memory burden and computation complexity, full CNNs (FCNNs), including 2D and 3D FCNNs serve as the backbone in many volumetric image segmentation tasks. In the biomedical segmentation application, U-Net is a well-behaved and efficient FCNN with a contracting path to capture context information and a symmetric expanding path for precise object localization. It can thus achieve the pixel-to-pixel semantic segmentation results^17^.

Although U-Net-based segmentation models can achieve relatively good segmentation performance in many biological tissue segmentation tasks, it is still intractable in the accurate segmentation of complex brain tumors. As concluded in many reports^7^ that brain tumor segmentation from MR images, especially the gliomas segmentation, is still a challenging task^6,16,18^.

Difficulties in accurate brain tumor segmentation and its 3D profile delineation mainly stem from (1) gliomas in MR images that may exhibit the same appearance with other tissues, e.g., gliosis; (2) gliomas structures that vary considerably across patients in terms of shape, appearance, size and so on; (3) unpredictable gliomas’s location, it may appear in any position in the brain; (4) gliomas usually has fuzzy or obscured boundaries due to its invasion to the surrounding tissue as well as the partial voluming or bias field artifacts; (5) there are intensity inhomogeneity in MR images; (6) brain tumors in MR images are usually noise-contaminated; (7) in addition, traditional segmentation methods are generally designed for the 2D image segmentation, which cannot achieve accurate 3D profiles of brain tumors.

It is well known that most medical data used in clinical practice are generally 3D voxel set^19^ (e.g. 3D CT, 3D MRI) and the medical diagnosis with surgical treatments have the requirement of accurate 3D structural characterization. Up to now, there are mainly two types of volumetric data processing methods. The first one involves converting the 3D volume into separate area, rather than as a whole. That is to say, processing the volumetric data slice by slice^20^ and sending each slice into a 2D image segmentation model firstly. Successively, the slice-based segmentation results are fused to generate the final 3D segmentation result. For instance, Zhao *et al*.^21^ proposed a method to obtain image blocks in axial, coronal and sagittal directions and input them into 2D CNN for the segmentation results fusion.

The other one involves directly devising a 3D CNN to detect and segment objects from volumetric data^22^. This can generally achieve better segmentation performance. For instance, Kumar *et al*.^23^ used the 3D CNN model to perform brain tumor segmentation. They extended the gray level co-occurrence matrix (GLCM) for features extraction and divided the discrete abnormal tissues from the raw data and GLCM feature space. Çiçek *et al*.^20^ proposed a 3DU-Net method for the semi-automatic and full-automatic segmentation of volumetric images.

To summarize, although many segmentation methods have achieved good segmentation results in the biological tissue segmentation tasks, there are still limitations in accurate brain tumor segmentation. Specifically, 2D CNN models generally ignore the context in the 3D profiles of objects in MR image volumes. The way of slice-based volumetric data processing method is relatively time-consuming and easy to lose the layer-wise context information of segmentation objects. Since these methods cannot make a full use of 3D context information. There is usually a lack of reliability and accuracy in object segmentation from volumetric data^16^. In addition, the direct use of 3D CNN (such as 3DU-Net) also shows limited adaptability when the network is not deep enough^20^, whereas the deep networks are computation intensive and prone to over-fitting. Moreover, the extremely unbalance between the tumor region and the background region will further deteriorate the brain tumor segmentation performance with traditional U-Net-like models. Therefore, this work attempts to devise a highly-efficient and reliable end-to-end automated 3D brain tumor segmentation model.

## Benchmark datasets

To make a quantitative assessment of the automated brain tumor segmentation models, benchmark dataset is also important for the segmentation model training and performance evaluation. As reviewed in Tiwari’s survey^24^, many datasets are available currently for training and test purpose, such as Internet Brain Segmentation Repository (IBSR)^25^, Cancer Genome Atlas GlioblastomaMultiforme (TCGA-GBM)^26^, Marmoset brain image dataset^27^, BRATS image dataset^2^, Digital Imaging and Communications in Medicine (DICOM) dataset^28^ and so on. These dataset are open access data sets, thus they do not require ethical committee approval.

In terms of the usage in the public literature, BRATS image dataset is the most widely-used benchmark dataset^29^. BRATS image dataset was created in 2012, when Menze *et al*.^2^ launched a Multimodal Brain Tumor Image Segmentation Benchmark (BRATS) challenge and then the open access benchmark dataset, BRATS image dataset, with MR images of low- and high- grade glioma patients with repeat manual tumor delineations by several human experts, as well as realistically generated synthetic brain tumor dataset with known ground truth, was published. Since 2013, BRATS datasets are update every year.

All BRATS multimodal scans are available as NIfTI files (.nii.gz) and describe a) native (T1) and b) post-contrast T1-weighted (t1Gd), c) T2-weighted (t2), and d) T2 Fluid Attenuated Inversion Recovery (Flair) volumes, and were acquired with different clinical protocols and various scanners from multiple (n = 19) institutions, mentioned as data contributors.

The BRATS 2013 provides clinical imaging data of 65 glioma patients, including 14 patients with low-grade gliomas (LGG) and 51 patients with high-grade gliomas (HGG). All images were skull stripped. The BRATS 2014, BRATS 2015, BRATS 2016, BRATS 2017 are all extensive version of their precedent versions. Generally, the follow-up version of BRATS image dataset include more variable appearances of brain tumors in the test set, with a greater challenge in the accurate brain tumor segmentation. Thus, in this work, the BRATS 2017 dataset is used for the performance validation of the proposed 3D brain tumor segmentation model. The publicly available BRATS dataset (RRID:SCR_016214) used in this paper can be found via the Google site link: https://www.med.upenn.edu/sbia/.

## Our contribution

Aiming at addressing the drawbacks from traditional FCNN-based brain tumor segmentation methods, this paper proposes an intersection over union (IOU) constraint 3D symmetric full convolutional neural network (IOUC-3DSFCNN) model with multimodal auto-context, to perform the end-to-end 3D brain tumor segmentation. By adding 3D residual groups to the classic 3DU-Net, IOUC-3DSFCNN has much deepened the network structure and can obtain more abstract features. Adapter blocks are incorporated in the IOUC-3DSFCNN model for the adoption feature mapping to promote the forward flow and back propagation of information. In order to obtain a comprehensive and end-to-end 3D brain tumor structure, the multimodal auto-context information is also introduced into the IOUC-3DSFCNN to extract accurate 3D brain tumor profiles. The main contributions of this paper are summarized as follows.

1. A novel 3D symmetric FCNN (3DSFCNN) model is proposed to perform an end-to-end 3D brain tumor segmentation, which transforms the traditional 2D slice-wise volumetric data processing and segmentation issue into an end-to-end 3D object segmentation task.
2. To address the issue of extremely unbalanced tumor segmentation labels in brain MR images, an IOU-constraint loss function is introduced to the proposed 3DSFCNN model to establish an IOUC-3DSFCNN model so as to further improve its 3D segmentation performance.
3. The multimodal auto-context information is incorporated into the IOUC-3DSFCNN model to improve the tumor detection and segmentation accuracy to assist in achieving the end-to-end 3D brain tumor profile.

The rest of the paper is arranged as follows. Section 2 details the structure of the proposed 3D brain segmentation model, including the basic architecture of the IOUC-3DSFCNN model and the multimodal auto-context fused IOUC-3DSFCNN framework. Detailed implementation of the proposed segmentation model is explained in Section 3. Section 4 details the confirmatory and comparative experimental results on benchmark BRATS 2017 dataset. Finally, Section 5 summarizes the whole paper and points out the potential further research directions.

## Methodology

This section mainly describes the architecture of the proposed 3D brain tumor segmentation model, including the architecture of the 3DSFCNN, the IOU constraint loss function and the multimodal auto-context fused 3DSFCNN framework.

### 3DSFCNN Architecture

**Figure 1:**
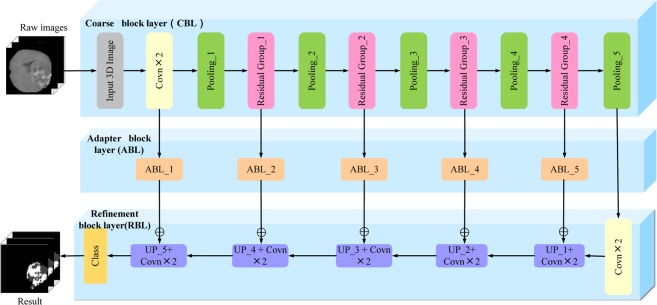
Schematic of 3DSFCNN. CBL, ABL and RBL are represented from top to bottom, respectively. CBL is used to extract features, RBL is used to restore the spatial information and the ABL is mainly used to adjust the forward flow and back propagation of network information.

Inspired by the classic 3DU-Net model, 3DSFCNN adopts a symmetrical cohesive network structure, which is composed of a coarse block layer (CBL), an adapter block layer (ABL) and a refinement block layer (RBL). Residual blocks are introduced into the 3DSFCNN to greatly deepen its network structure and prevent the gradient from vanishing^30^ during the model learning. Different from classic U-Net^31^ or FCNN^32^, adapter blocks are incorporated into the proposed network model to accelerate the forward flow and back propagation of network information. Schematic of the proposed 3DSFCNN model structure is shown in Fig. 1.

**(1) Coarse block layer (CBL)**. The CBL consists of 3D convolution layers, 3D max-pooling layers, and 3D residual groups. The application of convolutional layers aims at obtaining multiple feature maps from raw image with effective convolutional kernels, whose size are3 × 3 × 3. The calculation formula for the 3D convolution is defined as,

1 $${\rm{Y}}(m,n,l)=\mathop{\sum }\limits_{i=0}^{I-1}\mathop{\sum }\limits_{j=0}^{J-1}\mathop{\sum }\limits_{k=0}^{K-1}M(m+i,n+j,l+k)F(i,j,k)$$

where *M*(*) represents the raw MR image data, and *F*(*) represents the 3D convolution kernels.

The activation function is used to deal with nonlinear mapping. By adding some nonlinear factors to the 3DSFCNN model, the expressive and learning ability of the model can be enhanced. The activation function in this work is defined by

2 $$f(x)=\,\max (0,x)$$

The max-pooling operation is adopted in the segmentation model. The application of pooling layers can expand the receptive field with the deepening of the model structure. By the pooling operation, it can reduce the burden of raw data processing, thus decrease the computational time requirement. The convolution and pooling layer parameter settings are shown in Table 1.

**Table 1:** Convolution and pooling layer parameter setting.

Type	Filter size	Input	Output	Input filters	Output filters
Cov_1	3 × 3 × 3	128 × 128 × 128	128 × 128 × 128	4	32
Cov_2	3 × 3 × 3	128 × 128 × 128	128 × 128 × 128	32	64
Pooling_1	2 × 2 × 2	128 × 128 × 128	64 × 64 × 64	64	64
Pooling_2	2 × 2 × 2	64 × 64 × 64	32 × 32 × 32	128	128
Pooling_3	2 × 2 × 2	32 × 32 × 32	16 × 16 × 16	256	256
Pooling_4	2 × 2 × 2	16 × 16 × 16	8 × 8 × 8	512	512
Pooling_5	2 × 2 × 2	8 × 8 × 8	4 × 4 × 4	1024	1024
Cov_3	3 × 3 × 3	4 × 4 × 4	4 × 4 × 4	1024	2048
Cov_4	3 × 3 × 3	4 × 4 × 4	4 × 4 × 4	2048	4096
Cov_5	3 × 3 × 3	8 × 8 × 8	8 × 8 × 8	5120	1024
Cov_6	3 × 3 × 3	8 × 8 × 8	8 × 8 × 8	1024	512
Cov_7	3 × 3 × 3	16 × 16 × 16	16 × 16 × 16	1024	512
Cov_8	3 × 3 × 3	16 × 16 × 16	16 × 16 × 16	512	256
Cov_9	3 × 3 × 3	32 × 32 × 32	32 × 32 × 32	512	256
Cov_10	3 × 3 × 3	32 × 32 × 32	32 × 32 × 32	256	128
Cov_11	3 × 3 × 3	64 × 64 × 64	64 × 64 × 64	256	128
Cov_12	3 × 3 × 3	64 × 64 × 64	64 × 64 × 64	128	64
Cov_13	3 × 3 × 3	128 × 128 × 128	128 × 128 × 128	128	64
Cov_14	3 × 3 × 3	128 × 128 × 128	128 × 128 × 128	64	32

**(2) Residual group**. The residual network uses the input to add a quick connection to the output stacking layer, to increase the depth of the segmentation network model to retain more abstract feature information, to improve the MR image segmentation performance^33^.

In this work, four residual groups are used in the 3DSFCNN model, and each residual group includes three residual blocks. In order to fully reflect the performance advantage of the residual groups in the 3DSFCNN model, the 2D residual group is expanded into 3D residual group.

**Figure 2:**
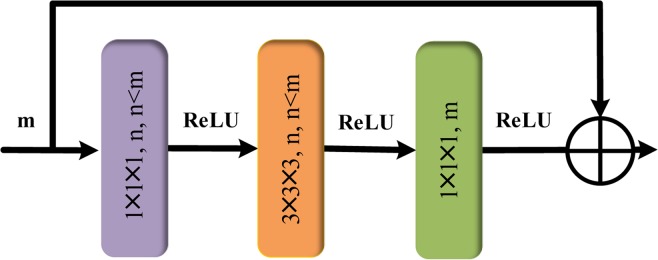
Schematic of residual block structure.

Schematic structure of the used residual block is shown in Fig. 2. The parameter settings of the residual groups are shown in Table 2. As can be seen from Fig. 2, the first layer and the third layer of the residual block adopt the 1 × 1 × 1 convolution kernel, and the middle layer adopts the 3 × 3 × 3 convolution kernel.

**Table 2:** Parameter settings of residual groups.

Type	Filter size	Input	Output
Res_1	$$[\begin{array}{c}(1\times 1\times 1)\\ (3\times 3\times 3)\\ (1\times 1\times 1)\end{array}]\times 3$$	$$[\begin{array}{c}(64\times 64\times 64)\\ (64\times 64\times 64)\\ (64\times 64\times 64)\end{array}]\times 3$$	$$[\begin{array}{c}(64\times 64\times 64)\\ (64\times 64\times 64)\\ (64\times 64\times 64)\end{array}]\times 3$$
Res_2	$$[\begin{array}{c}(1\times 1\times 1)\\ (3\times 3\times 3)\\ (1\times 1\times 1)\end{array}]\times 3$$	$$[\begin{array}{c}(32\times 32\times 32)\\ (32\times 32\times 32)\\ (32\times 32\times 32)\end{array}]\times 3$$	$$[\begin{array}{c}(32\times 32\times 32)\\ (32\times 32\times 32)\\ (32\times 32\times 32)\end{array}]\times 3$$
Res_3	$$[\begin{array}{c}(1\times 1\times 1)\\ (3\times 3\times 3)\\ (1\times 1\times 1)\end{array}]\times 3$$	$$[\begin{array}{c}(16\times 16\times 16)\\ (16\times 16\times 16)\\ (16\times 16\times 16)\end{array}]\times 3$$	$$[\begin{array}{c}(16\times 16\times 16)\\ (16\times 16\times 16)\\ (16\times 16\times 16)\end{array}]\times 3$$
Res_4	$$[\begin{array}{c}(1\times 1\times 1)\\ (3\times 3\times 3)\\ (1\times 1\times 1)\end{array}]\times 3$$	$$[\begin{array}{c}(8\times 8\times 8)\\ (8\times 8\times 8)\\ (8\times 8\times 8)\end{array}]\times 3$$	$$[\begin{array}{c}(8\times 8\times 8)\\ (8\times 8\times 8)\\ (8\times 8\times 8)\end{array}]\times 3$$

This kind of structure design can reduce the number of parameters to decrease the computational cost. We take the first convolution kernel of 1 × 1 × 1 to reduce the *m* channel data to *n* channel data, and then we restore the channel dimension to *m* by the convolution kernel of 1 × 1 × 1. With regard to the activation function, the rectifier linear units (ReLU) activation function instead of the sigmoid and tan his used in each residual block. The main reason is that the gradients of sigmoid and tanh are very gentle in the saturated region, leading to the gradient vanishing frequently and subsequently slowing down the convergence rate of the segmentation model.

**(3) Adapter block layer (ABL)**. ABL is mainly proposed for the feature fusion of CBL’s lower-dimensional feature maps and RBL’s high-dimensional feature maps. Specifically, we use the low-dimensional feature extracted from CBL to refine the high-dimensional feature in RBL. However, we must ensure that the feature maps we want to fuse have the same mapping size and channel number in advance. In this work, the bilinear interpolation is employed to improve the resolution of the low-resolution feature maps to that of the large feature maps that we want to fuse. On the premise that high-dimensional and low-dimensional feature maps have the same size, the corresponding two target feature maps are added pixel-wisely to perform the feature fusion. Thus, ABL can make two target feature maps with different sizes be consistent (as shown in Fig. 1, the output feature map size of the Residual Group_2 layer is 128 × 128 × 128, while the output feature map size of the UP_2 layer is 64 × 64 × 64) by the interpolation operation for the effective feature fusion.

**(4) Refinement block layer (RBL)**. This module is aimed at restoring the spatial information from 3D feature maps achieved by CBL in conjunction with the cohesion of context information. The pooling layers enlarge the receptive field of the extracted 3D feature maps. However, as the network deepens, the pooling operation will also miss partial feature information, which may lead to the issue of over-segmentation or under-segmentation. Therefore, we introduce a five-layer cohesion architecture into the 3DSFCNN model to restore the spatial information of the 3D feature maps. Bilinear interpolation is used to carry out up-sampling operation. Bilinear interpolation is an extension of a two-dimensional rectangular grid to interpolate bivariate functions such as *x* and *y*, expressed by

3 $$F({S}_{x,y})=[\begin{array}{cc}1-{S}_{x} & {S}_{x}\end{array}][\begin{array}{cc}F({S}_{0,0}) & F({S}_{0,1})\\ F({S}_{1,0}) & F({S}_{1,1})\end{array}]\,[\begin{array}{c}1-{S}_{y}\\ {S}_{y}\end{array}]$$

where $F({S}_{0,0})$, $F({S}_{0,1})$, $F({S}_{1,0})$ and $F({S}_{1,1})$ represents the pixel intensities of four known point coordinates at (0, 0), (0, 1), (1, 0) and (1, 1).

### IOU Constraint-3DSFCNN (IOUC-3DSFCNN)

In practical applications of brain tumor segmentation, the obtained brain tumor in MR images are often unbalanced (if the segmented region is taken as the foreground and the rest as the background, the foreground is only one thousandth of the backgrounds), which will result in a significant performance degradation of traditional segmentation models. There are mainly two ways to solve this problem. The first one involves re-sampling categories by extracting blocks (e.g., over-sampling or under-sampling) and the other one involves the use of a suitable loss function to optimize the network reasonably and focus on smaller targets (e.g., weighted cross entropy loss function, focal loss function).

The widely-used loss functions for the potential tackling of class imbalance involve weighted cross-entropy (WCE), DICE loss (DL), sensitivity-specificity (SS), generalized DICE Loss (GDL), and so on. As concluded in Sudre’s work^34^ that a middle or low imbalance task can be generally well handled by most of the loss strategies. However, for the 3D tumor segmentation task, it is a extreme imbalance situation. For a extremely unbalanced task, WCE is nearly unable to train, SS’s performance drops significantly, DL and GDL have the perform better than WCE and SS but they have very low learning rate^34^.

IOU, also known as Jaccard index, is the most commonly used metric for comparing the similarity between two arbitrary shapes, which encodes the shapes properties of the objects under comparison, e.g., widths, heights and locations, into the region property and then achieves a normalized measure that focuses on their volumes (or areas for 2D task) for object similarity evaluation. In the segmentation task, some efforts have been devoted to optimize IOU using either an approximate function or a surrogate loss and many studies have attempted to directly or indirectly incorporate IOU to achieve better segmentation performance^35,36^.

Inspired by Rezatofighi’s study^37^, an IOU constraint loss function is introduced as a substitute to the traditionally-used WCE or DICE loss function to generates an IOUC-3DSFCNN model toreduce the adverse influence of unbalance data in model training. The IOU constraint loss function can be formulated as

4 $${L}_{IOU}=1-\frac{1}{C}\frac{{\sum }_{c=1}^{C}\frac{1}{{\gamma }^{2}}{\sum }_{v=1}^{V}{X}_{cv}{Y}_{cv}}{{\sum }_{c=1}^{C}\frac{1}{{\gamma }^{2}}{\sum }_{v=1}^{V}({X}_{cv}+{Y}_{cv}-{X}_{cv}{Y}_{cv})}$$

where $\gamma =\mathop{\sum }\limits_{v=1}^{V}{Y}_{cv}$, defined as the weight of categories. ${Y}_{cv}$ is the ground truth (GT) category of $c$ at the $v{\rm{th}}$ pixel, ${X}_{cv}$ is the corresponding predicted probability value of category of $c$ at the $v{\rm{th}}$ pixel.

In the processing of unbalanced data, the small target, with some pixel prediction errors, will lead to a large change in the loss function. Subsequently, it will result in a sharp gradient change. Aimed at addressing the problem of unbalanced data, traditional methods mainly focus on small targets by weighting a few categories. The proposed IOU constraint loss function can reduce the influence on the number of model by weighting all categories in MR images, so as to improve the brain tumor (small object region) segmentation accuracy.

### Multimodal Auto-context Fused IOUC-3DSFCNN

In the field of medical image processing, different modalities of MR images can provide different texture boundary information, which are important visual perception clues^38^ for various image segmentation tasks. Therefore, the integration of multimodal MR image information will facilitate brain tumor segmentation. In this paper, four modalities of brain tumors are used in the brain tumor segmentation task, namely, t1, t1Gd, t2 and Flair. Inspired by Chen’s work^39^, multimodal MR images are used as inputs to the IOUC-3DSFCNN model to obtain the probability maps of brain tumors and the weight of each modality.

**Figure 3:**
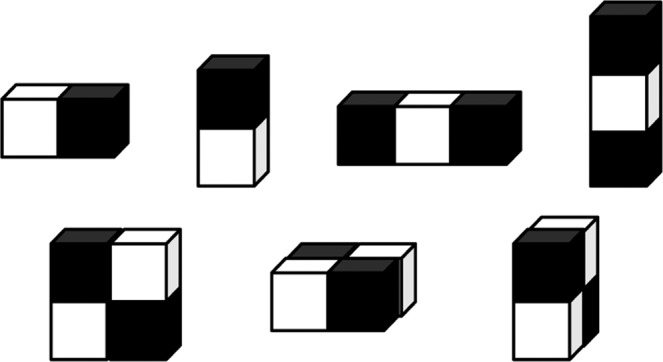
3D Haar-like features.

The Haar-like feature mentioned in the literature^40^ is also incorporated for the feature fusion to facilitate the 3D brain tumor segmentation task. Haar-like feature extraction is a very classic feature extraction method. Haar-like feature is generated by a series of feature templates involving edge feature, linear feature, center feature and diagonal feature. Only white and black cuboids are in the feature template, and the feature values of templates are defined as the difference between the white cuboid pixels and the black cuboid pixels. Since volumetric MR images are used in this work, the traditional 2D Haar-like feature is extended to 3D Haar-like feature. In order to save calculation cost, the integral volumetric method is used to calculate the 3D Haar-like feature value efficiently. Examples of 3D Haar-like feature templates are shown in Fig. 3.

In order to make a full use of the integration of the context information, multimodal features complementary information and 3D Haar-like features, we propose an auto-context-fused segmentation method. Traditional auto-context algorithms learn a series of classifiers by combining context information with raw image appearances^40^. Given a set of training set data $S=\{{S}_{1},{S}_{2},\mathrm{..}.,{S}_{n}\}$ with its corresponding label $Y=\{{Y}_{1},{Y}_{2},\mathrm{..}.,{Y}_{n}\}$, it firstly learns a classifier based on local image patches. The discriminative probability maps generated by the learned classifier are then used as the context information to train a new classifier in conjunction with the original image patches. Auto-context integrates low-level and context information by fusing a large number of low-level appearance features with context and implicit shape information. Compared with recognition tasks in natural image processing, auto-context information may play a more significant role in the medical field due to the complexity of brain structure^40^.

**Figure 4:**
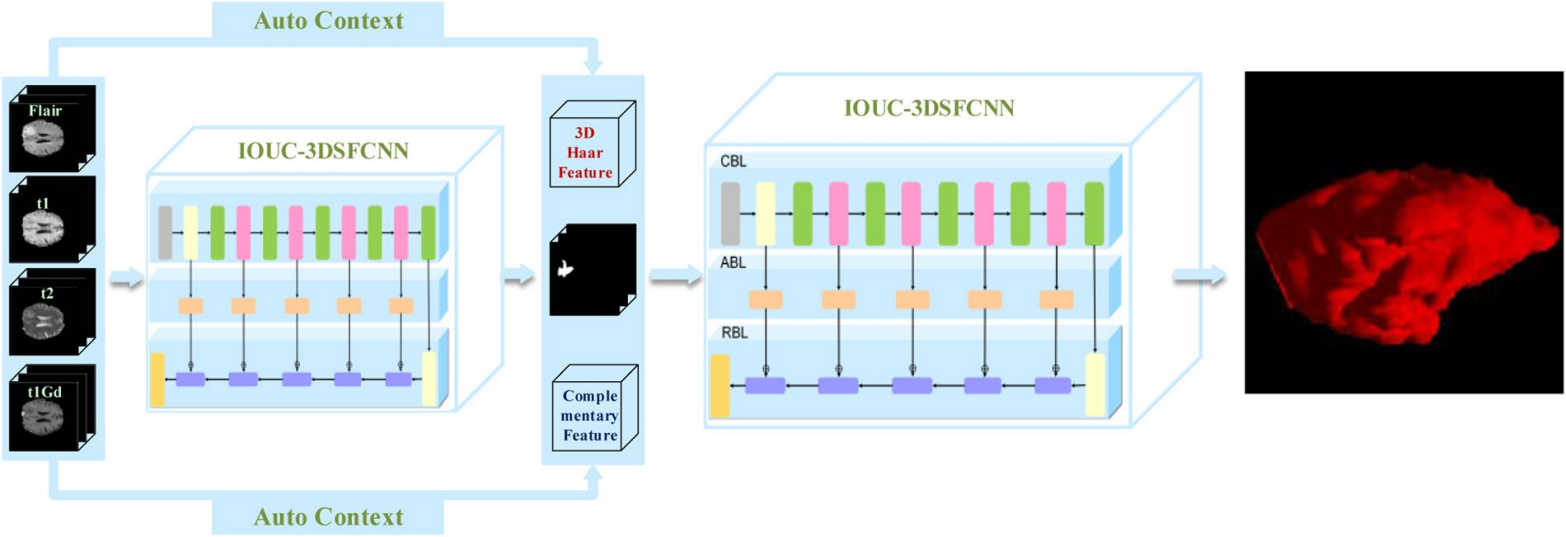
Schematic of the proposed multimodal auto-context fused IOUC-3DSFCNN.

To summarize, in contrast with methods described in literature^39,40^, a new network model (IOUC-3DSFCNN) is adopted as the classifier for brain tumor segmentation. In addition, 3D Haar-like features and complementary feature information extracted from the raw images are combined with the probability maps generated by the IOUC-3DSFCNN-based classifier to train a new classifier. Then, the auto-context fused IOUC-3DSFCNN model is established and its schematic is displayed in Fig. 4.

As can be seen in Fig. 4, given the training dataset, we firstly train an IOUC-3DSFCNN classifier on original MR images. Then, the discriminative probability maps generated from an IOUC-3DSFCNN classifier are used as the context information, in combination with the 3D Haar-like features and complementary feature information extracted from the raw images are used as the input of another IOUC-3DSFCNN-based classifier model, which further refines the segmentation results. Consistent with the view of Chen *et al*.^39^, the result of the second classifier is adopted as the final segmentation result, instead of the iterative method.

### Post-processing

Post-processing is employed in an attempt to solve the problem of misclassified voxels. We regard the tumor with small connected area as misclassified voxels, which will be ignored by a threshold method, i.e., delete the false tumor region with the connected area less than a predefined threshold. The predefined threshold is set as one tenth of the maximum connected area adaptively.

## Experimental Set Up

### Data acquisition and pre-processing

#### Data acquisition

**Figure 5:**
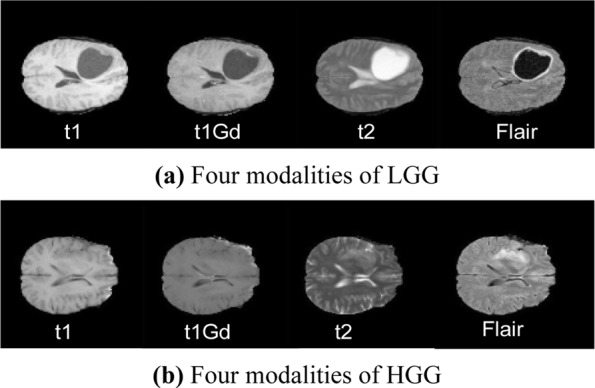
Four modalities of HGG and LGG. From left to right: t1, t1Gd, t2, and Flair.

The BRATS 2017 dataset used in this work involves 167 glioma samples, including 102 samples of glioblastoma (HGG) and 65 samples of lower grade glioma (LGG). Four brain tumor modalities are included in each glioma sample, namely, the original state (t1), t1-weighted (t1Gd), t2 and liquid decay inversion recovery (Flair). In addition, the LGG has clear and smooth contours, as indicated in Fig. 5(a). HGG shows infiltrating growth with no regular shape of edge contour, as indicated in Fig. 5(b). All datasets are clinically normal for 3 T multi-peak MRI scan function, all ground truth labels are manually revised by neuropathologists certified by the expert committee, and the labels are divided into five categories: healthy tissue (label 0), necrotic tissue (label 1), edema tissue (label 2), non-enhanced tissue (label 3) and enhanced tumor tissue (label 4).

#### Data Pre-processing

In the image segmentation task, data augment is very important because it can effectively improve the robustness of a machine learning model^41^. The acquired dataset is divided into training set and test set, where the training set includes 80% samples and the remaining samples consist of the test set. Specifically, a total of 167 four modalities MR image data with segmentation label are obtained in the experiments, with a total of 167 × 4 = 668 data samples with the image size of 240 × 240 × 155.

Data augment processing is carried out to enable model training to achieve a more reliable segmentation model. The 180-degree rotation data expansion technology is used without any mapping change of the image’s label value, so the training set and test set after data augment have 1068 and 268 samples, respectively. Moreover, the N4ITK method proposed by Zhou *et al*.^42^ is also adopted to correct the effect of the bias field. The pre-processing steps are as follows.

Step 1. Rotate the input sample image data with 180 degrees.

Step 2. The N4ITK bias correction is applied to each sampling image data.

Step 3. Each input sampling MR image data is normalized by subtracting the average of all pixels from each pixel in the image and dividing by the standard deviation.

### Parameter settings

In this paper, the multimodal auto-context fused IOUC-3DSFCNN model is established for the brain tumor segmentation. Model parameters are set as follows. In order to ensure the objective function convergenceto the local minimum value in an appropriate time, the learning rate is initialized to 0.0001. If the loss value does not decrease during the training process, the learning rate is halved. The second optimization parameter is the number of iterations for model training. Since the manual adjustment of the iteration number is time-consuming, we initialize the iteration number to be 100 at the beginning and set a fixed parameter of the error rate. When the error rate is equal to this parameter, the training will be stopped and the current number of iterations is considered as an appropriate number. In addition, in order to prevent the network model from over-fitting in the training process, the dropout operation is also used for model optimization, and the dropout value is set at 0.5. Experimental results demonstrated that the abovementioned parameter setting can optimize the performance of the proposed segmentation model, i.e., effectively improve the segmentation efficiency and accuracy of the whole brain tumor segmentation model.

### Implementation

Our method was implemented in Python under theTensorflow framework, and the computation was performed under the hardware of NVIDIA GTX 1070 GPU. Due to the limited capacity of GPU memory, we cropped each subject (size 240 × 240 × 155) into sub-volumes (size 128 × 128 × 128) to remove the black areas at edges from the input to the segmentation network. In order to enlarge the receptive field, the max-pooling operation with the pooling kernel of 2 × 2 × 2 is used for down-sampling in the abstract feature extraction (in the CBL of the 3DSFCNN). The training time of the whole training set is about 48 hours, and the accuracy rate is 0.8426.

## Experimental Results and Discussion

### Performance evaluation criterion

In this work, DICE similarity coefficient (DICE), Recall,Precision and Hausdorff distance^43^ are used to evaluate the segmentation performance. $DICE$ indicator is defined as

5 $$DICE=\frac{2|T\cap P|}{T\cup P}$$

where $T$ represents the ground truth; $P$ represents the predicted result value. The closer the DICE to 0, the worse the segmentation effect. Re *call* indicator is defined as

6 $$\mathrm{Re}\,call=\frac{TP}{TP+FN}$$

where *TP* is true positive and ${FN}$ is the false negative result. The closer the Recall to be 1, the better the segmentation effect. Pr *ecision* indicator is defined as

7 $$\Pr \,ecision=\frac{TP}{TP+FP}$$

similarly, the closer the Precision is to 1, the better the segmentation effect. The Hausdorff distance is defined as

8 $$HD=\,\max \,\{hd(P,T),hd(T,P)\}$$

9 $$hd(P,T)=\mathop{\max }\limits_{p\in P}\mathop{\min }\limits_{t\in T}\Vert p-t\Vert $$

10 $$hd(T,P)=\mathop{\max }\limits_{p\in P}\mathop{\min }\limits_{t\in T}\Vert t-p\Vert $$

where $T$ represents the ground truth;$P$ represents the predicted value, p and t are points on P and T, respectively; $\Vert \bullet \Vert$is a form of distance. In this work, the Euclidean distance is used.

### Experimental results

#### Effectiveness of multimodal auto-context fused IOUC-3DSFCNN

In this section, the feasibility of the multimodal auto-context fused IOUC-3DSFCNN method is primarily validated and it shows higher complexity and variability of brain tumors than its earlier benchmark editions, such as BRATS 2013, BRATS 2015, and so on^44^. The proposed method is applied to the brain tumor lesion area segmentation of patients on BRATS 2017 dataset for the segmentation performance evaluation.

**Figure 6:**
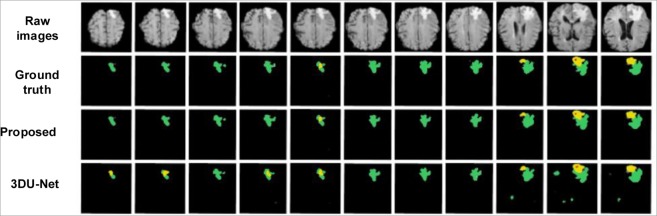
Experimental results of subject 1. From top to bottom are raw images, ground truth and segmentation results of the proposed method, 3DU-Net proposed by Çiçek *et al*.^20^, respectively.

**Figure 7:**
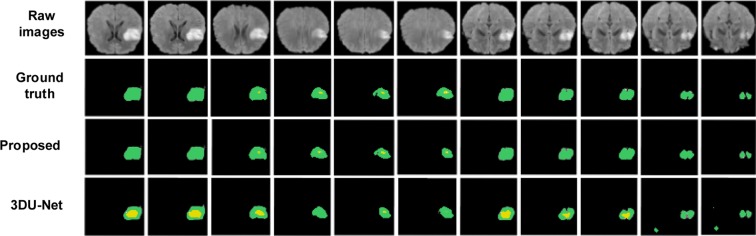
Experimental results of subject 2. From top to bottom are raw images, ground truth and segmentation results of the proposed method and 3D U-Net (proposed by Çiçek *et al*.^20^), respectively.

Figures 6 and 7 show the segmentation results with corresponding ground truth of the brain tumor lesion area of two patients, respectively (Figs. 6 and 7 only show the results of partial slices). It is observed in Figs. 6 and 7 that the segmentation results of lesion areas achieved by the proposed segmentation model are close to the ground truth. In other words, it is demonstrated that the proposed multimodal auto-context fused IOUC-3DSFCNN method has higher localization accuracy and more reliable segmentation ability.

**Figure 8:**
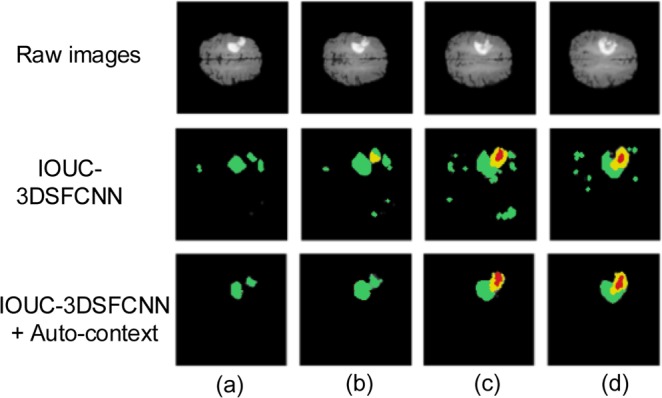
Segmentation result comparison. (**a–d**) represent four different slices of the same brain tumor.

To further evaluate the effectiveness of the multimodal auto-context fusedIOUC-3DSFCNN model, we have conducted more validation experiments on BRATS 2017 dataset. Table 3 lists the evaluation results of the 3DSFCNN model and the multimodal auto-context fused IOUC-3DSFCNN model. It can be seen from Table 3 that the proposed multimodal auto-context fused IOUC-3DSFCNN can achieve higher quantitative evaluation indexes, DICE, Recall and Precision. Some visual segmentation results are shown in Fig. 8. As seen in Fig. 8, the performance of the auto-context fused IOUC-3DSFCNN method is significantly better than the IOUC-3DSFCNN model. The proposed method overcomes the difficulty of blurring brain boundaries and itcan effectively extract 3D brain tumor structures from complex MR images.

**Table 3:** Performance comparison of the proposed multimodal auto-context fused IOUC-3DSFCNN model with 3DSFCNN model.

Methods	DICE			Recall			Precision			HD(mm)		
	Whole	Core	Enh.	Whole	Core	Enh.	Whole	Core	Enh.	Whole	Core	Enh.
IOUC-3DSFCNN	0.82	0.81	0.70	0.88	0.80	0.79	0.84	**0.86**	0.74	7.63	8.31	9.08
IOUC-3DSFCNN + Auto-context	**0.89**	**0.84**	**0.78**	**0.91**	**0.84**	**0.85**	**0.88**	0.85	**0.78**	**4.03**	**6.32**	**7.85**

**Figure 9:**
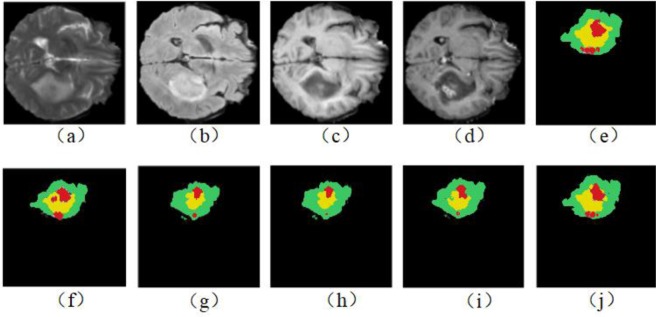
Segmentation results using different modal MR images. (**a–d**) represent the raw images of four single modalities; (**e**) represents the ground truth, (**f–i**) represent the segmentation results of Flair, t1, t1Gd, and t2 modality, and (**j**) represents the segmentation result of the multimodal MR images.

We have also compared the performance based on four single-modalities and multimodality (including t1, t1Gd, t2 and Flair) on the BRATS 2017 dataset to verify the effectiveness of multimodal information. Table 4 lists the performance evaluation results of single-modality and multimodality. Some visual brain tumor segmentation results are shown in Fig. 9.

**Table 4:** Performance comparison using different modalities.

Modalities	DICE			Recall			Precision			HD(mm)		
	Whole	Core	Enh.	Whole	Core	Enh.	Whole	Core	Enh.	Whole	Core	Enh.
Flair	0.80	0.77	0.68	0.82	0.80	0.71	0.85	0.83	0.71	6.55	7.16	9.44
t1	0.79	0.65	0.56	0.75	0.78	0.63	0.77	0.55	0.61	7.31	12.19	15.67
t1Gd	0.81	0.80	0.74	0.85	0.80	0.75	0.81	0.79	0.75	5.76	7.47	9.29
t2	0.84	0.79	0.80	0.84	0.74	0.72	0.86	0.84	0.73	5.88	6.33	8.90
Flair+t1+t1Gd + t2	0.88	0.84	0.78	0.89	0.85	0.79	0.85	0.86	0.76	4.28	7.94	7.83

As can be seen from Table 4, performance indicators of the t1 modality are relatively low, and multimodalities-based segmentation performance are much better in terms of almost all the evaluation indicators. This is mainly because different modalities are beneficial to different tissues characterization and presumably t1 modality contributes to the differentiation of normal tissue and weakens the characteristics of tumors. Therefore, the t1 modality is conducive to the segmentation of normal tissue but unprofitable to the brain tumor segmentation. By incorporating the multimodal MR images, different tissues can be delineated effectively. Thus, the multimodal MR images-based tumor segmentation can achieve better segmentation results.

It can be observed from Fig. 9 that there are under-segmentation in the single-modality, and the results of multimodal brain tumor segmentation are closer to the ground truth. Therefore, in terms of the visual segmentation results in Fig. 9 and the numeric evaluation indicators listed in Table 4, the full utilization of complementary information under different modalities can effectively improve brain tumor segmentation accuracy.

#### Comparison with Classic 3DU-Net

**Figure 10:**
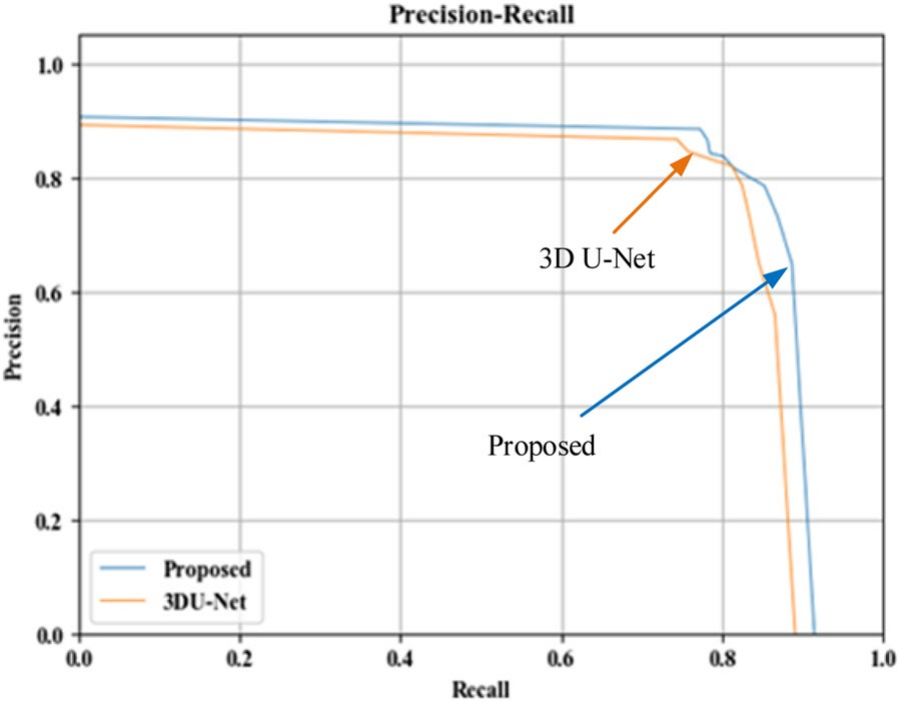
P-R curve comparison.

To further evaluate the segmentation performance of the proposed method, we compared the proposed method with the classic 3DU-Net model. Firstly, the generalization performance of the proposed segmentation model is analyzed using P-R curve, displayed in Fig. 10. The P-R curve is precision and recall curve, with recall as the horizontal axis and precision as the vertical axis.

It can be seen from Fig. 10 that the P-R curve of the proposed brain tumor segmentation method is obviously superior to the 3DU-Net model, indicating that the generalization performance of the proposed auto-context fused IOUC-3DSFCNN model is better than that of the classic 3DU-Net model. As can be seen from Fig. 6 and Fig. 7, where the last row displays the slices of the prediction results of 3DU-Net model proposed by Çiçek^20^, the segmentation effect of the proposed method is visually better than that of the 3DU-Net. The classic 3DU-Net model shows some over-segmentation in the brain tumor segmentation.

In Table 5, an objective evaluation indicators of the 3DU-Net model and the proposed method are listed explicitly. Apparently, as shown in Table 5, the proposed method offersapparently higher values on the DICE, Recall and Precision indicators than the 3DU-Net model.

**Table 5:** Comparison with 3DU-Net brain tumor segmentation method.

Methods (with multimodality)	DICE			Recall			Precision			HD(mm)		
	Whole	Core	Enh.	Whole	Core	Enh.	Whole	Core	Enh.	Whole	Core	Enh.
**Proposed**	**0.89**	**0.84**	**0.78**	**0.91**	**0.84**	**0.85**	**0.88**	**0.85**	**0.78**	**4.03**	**6.32**	**7.85**
**3DU-Net**	0.87	0.83	0.77	0.90	0.80	0.84	0.84	0.85	0.75	7.57	10.44	13.06

The proposed brain tumor segmentation model deepens the network model of the classic 3DU-Net by adding 3D residual groups on a five-layer cohesion architecture-based adapter block layertorefine the 3D feature maps by fusing low level abstract information. The residual network can alleviate the problem of gradient disappearance in the deep neural network. In addition, the IOU constraint with multimodal auto-context framework can achieve more precise tumor segmentation delineation results due to the information complementation from the context information and multimodality MR images.

In summary, the proposed multimodal auto-context fused IOUC-3DSFCNN fully uses the advantages of residual network, and benefits from the IOU constraint and multimodal auto-context framework, and can therefore detect and extract the 3D structure of various brain tumors in complex fuzzy brain tumors from MR images.

#### Comparison with other state-of-the-art methods

To make a comprehensive comparison with the latest brain tumor segmentation methods, the experimental prediction results of some state-of-the-art models are listed in Table 6, where the experimental results are all the best reported values in theiroriginatedliterature. As listed in Table 6, Hu *et al*.^16^, Pereira *et al*.^5^, Razzak *et al*.^45^, Yang *et al*.^46^, Zhao *et al*.^21^ and Xue *et al*.^47^ all used 2D CNNs to perform brain tumor segmentation. Sun *et al*.^48^ used a kind of 3DU-Net-like segmentation model.

**Table 6:** Performance comparison of other brain tumor segmentation methods.

Methods (with multimodality MRI)	Evaluation											
	DICE			Recall			Precision			HD(mm)		
	Whole	Core	Enh.	Whole	Core	Enh.	Whole	Core	Enh.	Whole	Core	Enh.
**Proposed**	0.89	**0.84**	0.78	0.91	0.84	0.85	0.88	0.85	0.78	**4.03**	6.32	7.85
Hu *et al*.^16^	0.89	0.82	0.77	0.90	0.84	0.86	0.87	0.83	0.71	—	—	—
Pereira *et al*.^5^	0.88	0.83	0.77	0.89	0.83	0.81	0.88	0.87	0.74	—	—	—
Razzak *et al*.^45^	0.89	0.79	0.75	0.88	0.80	0.80	0.88	0.87	0.68	—	—	—
Yang *et al*.^46^	**0.90**	0.82	**0.87**	**0.93**	**0.88**	**0.93**	0.88	0.78	**0.83**	—	—	—
Zhao *et al*.^21^	0.88	0.84	0.77	0.86	0.82	0.80	**0.90**	**0.87**	0.76	—	—	—
Sun *et al*.^48^	0.84	0.72	0.62	0.89	0.73	0.69	0.82	0.77	0.60	—	—	—
Xue *et al*.^47^	0.85	0.70	0.66	0.80	0.65	0.62	0.92	0.80	0.69	—	—	—
Isensee *et al*.^49^	0.89	0.79	0.73	0.89	0.78	0.79	—	—	—	6.97	9.48	4.55
Chen *et al*.^52^	0.83	0.73	0.64	0.84	0.74	0.80	—	—	—	36.4	25.59	30.31
Wang *et al*.^50^	0.87	0.77	0.78	—	—	—	—	—	—	6.55	27.04	15.90
Wang *et al*.^51^	0.87	0.79	0.74	—	—	—	—	—	—	4.16	5.97	6.71

Comparative results in Table 6 reveal that the improved U-Net baseline model proposed by Yang *et al*.^46^ is well suitable for the brain tumor segmentation task, and can achieve a relatively optimized model structure with feature recombination layers to the baseline model. Thus, the baseline U-Net model proposed by Yang^46^ achieve the state-of-the-art brain tumor segmentation performance, namely, it achieve higher values of DICE, Recall and Precision than these comparative methods. The proposed multimodal context fused IOUC-3DSFCNN model can achieve comparable results. In other words, the state-of-the-art performance by the baseline U-Net model is only slightly higher than the proposed multimodal context fused IOUC-3DSFCNN model in terms of the evaluation indicators. Moreover, the proposed method can even achieve superior results on some indicators than the baseline U-Net model.

Isensee *et al*.^49^, Wang *et al*.^50,51^ and Chen *et al*.^52^ have also conducted experiments based on the BRATS 2017 dataset and used HD indicators for evaluation. As can be seen from Table 6, the proposed method can approach nearly the best indicators among these comparative methods. To summarize, the proposed segmentation method in this paper has a higher performance on the brain tumor segmentation than these state-of-the-art methods.

### Discussion

The proposed multimodal auto-context fused IOUC-3DSFCNN model is inspired by the classic U-Net model and the auto-context techniques proposed by Tu and Bai^40^. However, the model structure of the proposed IOUC-3DSFCNN is different from the classic 3DU-Net or FCNN models. It involves a coarse block layer (CBL), an adapter block layer (ABL) and a refinement block layer (RBL). Extensive experiments have demonstrated that the proposed method is more suitable for MR image segmentation tasks even with blurred tumor boundaries.

The proposed segmentation model combines multimodal MR image features, 3D Haar-like features, and tumor probability map to generate a multimodal auto-context fused IOUC-3DSFCNN model, used to capture the spatial structuremaps of different tissues in MR images and extract the 3D brain tumor profile. The IOU constraint loss function is employed to make up for the region imbalance of different tissues in MR images. At the beginning of the 3DSFCNN model design, we attempted touse a deeper FCNN model to perform the tumor segmentation, but experimental results showed that the segmentation effect was not obviously superior, and there was a phenomenon of over-segmentation, which may mainly result from the gradient disappearance or gradient explosion as the network deepens in the model learning. Considering the characteristics and advantages of the residual network, 3D residual groups are adopted to address the problem of gradient vanishing.

To summarize, the proposed segmentation model can achieve good segmentation performance mainly due to the following three aspects: The first one is the network hyper-parameter optimization. One hyper-parameter is the learning rate. We have conducted considerable experiments to find the optimal learning rate to facilitate the effective brain tumor segmentation. Another parameter is the number of iterations. We set a threshold for error rate monitoring during the model training, when the performance of the model is not raised significantlyor becoming stable, the number of iterations is terminated. The optimization of the above two parameters improves the generalization performance of the proposed network model. The second one is that an IOU constraint loss function is employed to address the problem of unbalanced tissues regions so to prevent the model deterioration of tumor segmentation. The last is that the proposed framework of multimodal auto-context IOUC-3DSFCNN is proposedtakes full account of the multimodal information in the 3DMR image space context.

However, it can be seen from the comparative experiments that the proposed segmentation model still has certain limitations. Firstly, in the model training, it still exist some issues, such as high computational complexity with high memory burden. Due to limited computer performance, we reduced the original image size to achieve efficient model training. Hence, it will be necessary to further study the dimensionality reduction or more efficient methods in future, and reduce the calculation cost on the basis of accuracy improvement. The second limitation is that only one dataset is used for the performance validation and comparison. More datasets with richer experimental objects should be used to assess the model performance for further model optimization and achievement of reliable and efficient segmentation results.

## Conclusions

In brain tumor segmentation using MR images, over-segmentation or under-segmentation problems are frequent due to cross interference of scanning instrument and fuzzy boundaries of brain tumors. In this paper, a multimodal auto-context fused IOUC-3DSFCNN brain tumor segmentation method is presented. The proposed 3DSFCNN model generates probability maps by learning features of HGG and LGG MR images, combining 3D Haar-like features with multimodal complementary features to obtain 3D brain tumor profiles that are not affected by boundary ambiguity. Extensive confirmatory and comparative experiments show that the proposed method can extract features of brain tumors in complex environments, and can thus achieve effective 3D brain tumor segmentation results, hence laying a reliable foundation for medical diagnosis, treatment planning and postoperative measures of brain tumors. Further work should focus on combing multi-tasking learning thoughts so as to achieve end-to-end aggressive type classify or survival prediction with 3D brain tumor location, segmentation and profile characterization.
